# Malignant triton tumor of the anterior mediastinum: A case report

**DOI:** 10.3892/ol.2014.1787

**Published:** 2014-01-09

**Authors:** WEI REN, XINYUN XU, JING YAN, XIAOPING QIAN, BAORUI LIU

**Affiliations:** 1 Department of Oncology, The Affiliated Drum Tower Clinical Medical College of Nanjing Medical University, Nanjing, Jiangsu 210008, P.R. China; 2 The Comprehensive Cancer Center of Drum Tower Hospital, Medical School of Nanjing University and Clinical Cancer Institute of Nanjing University, Nanjing, Jiangsu 210008, P.R. China; 3 Department of Pathology, Drum Tower Hospital, Medical School of Nanjing University, Nanjing, Jiangsu 210008, P.R. China

**Keywords:** malignant triton tumor, malignant peripheral nerve sheath tumor, mediastinum

## Abstract

Malignant triton tumors (MTTs) are a rare subtype of malignant peripheral nerve sheath tumor (MPNST) showing rhabdomyoblastic differentiation, which have no treatment consensus and a poor prognosis. This case report presented the case of a 42-year-old male patient with a large MTT located in the anterior mediastinum. The patient underwent palliative chemoradiotherapy and interstitial chemotherapy and received traditional Chinese medicine. Localization of an MTT in the anterior mediastinum is extremely rare. To the best of our knowledge, this is only the fourth study of an MTT localized in the anterior mediastinum that has been documented in the English literature.

## Introduction

Malignant peripheral nerve sheath tumors (MPNSTs) are believed to arise from Schwann cells or nearby cells with perineural differentiation. The incidence of MPNSTs is one per 100,000 cases. In total, ~50% of MPNST cases arise in patients with neurofibromatosis type 1 (NF1; von Recklinghausen’s disease) and the five-year overall survival rate is 34% (1). Malignant triton tumors (MTTs) are a subgroup of MPNSTs, which display rhabdomyosarcomatous differentiation and follow a particularly aggressive course. MTTs account for <10% of MPNSs, and are identified by focal evidence of skeletal muscle differentiation within the MPNSTs. Positive immunohistochemical staining for desmin, actin and myogenin is evidence of skeletal muscle differentiation (1). A recent study reported that the five-year survival rate and median survival time of patients with MTT were 14% and 13 months, respectively (2). To date, >100 cases of MTTs have been reported in the English literature (2). Regarding the original location, MTTs occur predominantly in the head, neck and trunk regions (3). Only eight cases have previously been observed in the mediastinum (Table I) (3–10). The current study presents the case of a 42-year-old male patient with an MTT arising from the anterior mediastinum. To the best of our knowledge, this is the fourth report that has been documented in the English literature worldwide regarding an MTT localized in the anterior mediastinum. Written informed consent was obtained from the patient.

## Case report

**Figure 1:**
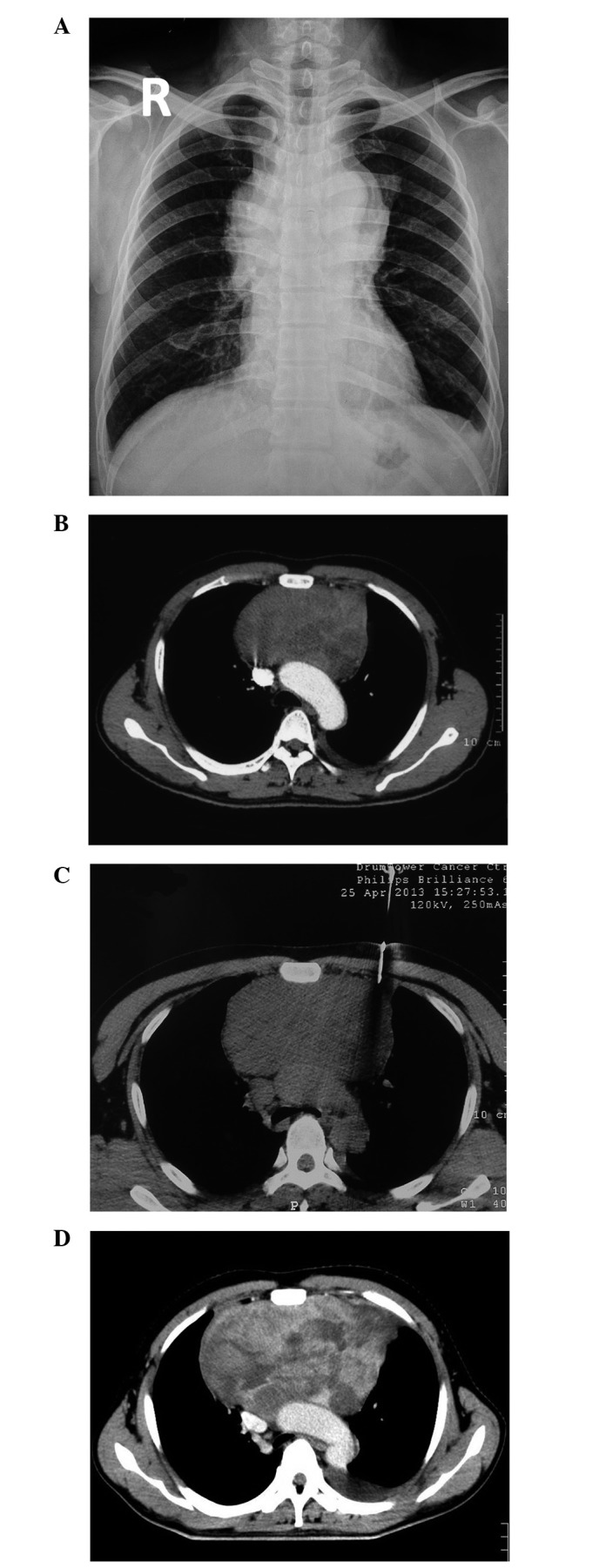
(A) Chest radiography revealied the mediastinal mass on April 23, 2013. (B) Chest computed tomography (CT) revealed a large anterior mediastinal tumor invading the great vessels on April 23, 2013. (C) A percutaneous core cutting needle biopsy of the anterior mediastinal tumor was performed under CT guidance. (D) Chest computed tomography re-examination revealed that the anterior mediastinal tumor was enlarged 1 month after the initial CT scan (May 23, 2013).

A 42-year-old male patient with a history of a dry cough for 2 months and chest distress for 1 month was treated at the clinic of Zhangjiagang Bo’ai Hospital (Suzhou, China) on April 23, 2013. The results of the chest radiograph (Fig. 1A) and enhanced computed tomography (CT) scan (Fig. 1B) revealed a large anterior mediastinal mass, measuring 14.8×12.0×11.5 cm in size and invading the mediastinal great vessels. The patient was then admitted to Nanjing Drum Tower Hospital (Nanjing, China) for further assessment and therapy. In addition, a percutaneous core cutting needle biopsy of the anterior mediastinal mass was performed with CT guidance (Fig. 1C).

### Pathological diagnosis of an MTT

**Figure 2:**
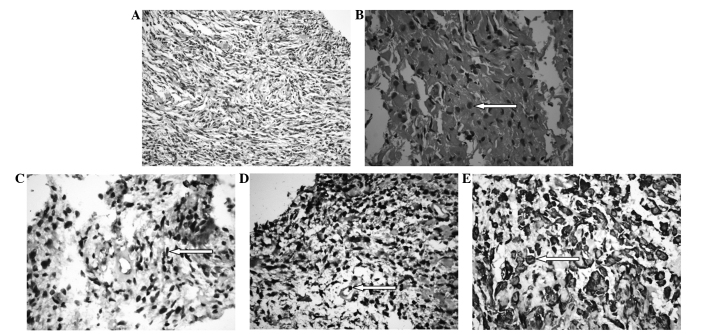
(A) Malignant peripheral nerve sheath tumor (MPNST) cells. Malignant spindle cells with marked pleomorphism and fasciculated architecture were observed (hematoxylin and eosin; magnification, ×200). (B) High-powered view of MPNSTs with rhabdomyosarcomatous differentiation. Round cells with eosinophilic cytoplasm were morphologically consistent with rhabdoid differentiation and were identified in a background of classic MPNST (white arrow) (hematoxylin and eosin; magnification, ×400). (C) Positive nuclear immunohistochemical staining with S-100 was noted in MPNST (white arrow) (magnification, ×400). (D) Positive nuclear immunohistochemical staining with myogenin was noted in rhabdomyoblastic cells (white arrow) (magnification, ×400). (E) Positive cytoplasm immunohistochemical staining with desmin was noted in rhabdomyoblastic cells (white arrow) (magnification, ×400).

Histological analysis revealed a spindle cell tumor with interlacing fascicles of wavy spindle cells and prominent mitotic figures. Loose and impact arrangements were detected. In certain focal areas, the tumor was comprised of round rhabdomyoblasts, with abundant eosinophilic cytoplasm and eccentric nuclei in a loose matrix. Immunohistochemical analysis revealed that the spindle cells were positive for S-100, and the rhabdomyoblasts were positive for myogenin and desmin, respectively (Fig. 2). Simultaneously, the tumor cells were negative for neuron-specific enolase, B-cell lymphoma 2, cytokeratin 19, cluster of differentiation (CD)57, myogenic regulatory protein, neuronal nuclear antigen and smooth muscle actin.

Considering the locally advanced staging of the anterior mediastinal MTT, surgical excision was ruled out. Chest computed tomography re-examination revealed that the anterior mediastinal MTT had enlarged rapidly to 17.0×15.0×12.0 cm on May 23, 2013 (Fig. 1D). Palliative intensity-modulated radiotherapy with concurrent intravenous and interstitial chemotherapy using liposomal paclitaxel was performed. The patient remains alive with the disease and is currently receiving traditional Chinese medicine.

## Discussion

In 1932, Masson (11) was the first to describe a neurogenic tumor accompanied by rhabdomyoblasts. In 1973, Woodruff *et al* introduced the term MTT in a review of seven cases (12). The majority of pathologists use the term MTT to refer to tumors that exhibit the features of MPNSTs and contain rhabdomyoblastic elements, no matter what their location (3).

Malignant tumors arising from Schwann cells of peripheral nerves or within existing neurofibromas are collectively referred to as MPNSTs. MTTs are a subgroup of MPNSTs, which have been reported to display rhabdomyosarcomatous differentiation and follow a particularly aggressive course (13). MTT accounts for <10% of MPNSTs and is identified by focal evidence of skeletal muscle differentiation within a MPNST. NF1 (von Recklinghausen’s disease) has been associated with an increased risk of MPNST and MTT. In total, ~69% of MTT cases are diagnosed in patients with von Recklinghausen’s disease and occur in young male patients, whereas the remaining 31% are sporadic cases mostly occurring in older females (3). The results of previous studies have differed with regard to the impact of NF1 on the prognosis of patients with these tumors, although the general consensus is that NF1 is a negative prognostic factor. In the present study, the patient with anterior mediastinal MTT did not suffer from von Recklinghausen’s disease.

The cell of origin of MTT remains unclear, although the presence of neural cells and rhabdomyoblasts has led certain authors to hypothesize that these two cell components were derived from less-differentiated neural crest cells. These cells have mesodermal and ectodermal differentiation potential and thus possess the ability to develop skeletal and neural components. Direct evidence for the potential of schwannoma cells to exhibit myogenic differentiation has been previously shown (14). Immunohistochemical staining aids the identification of the origin of cells. Nerve sheath differentiation is confirmed by S-100 protein and Leu-7 (CD57) positivity, whereas rhabdomyoblastic differentiation is confirmed by positivity to desmin, actin and myogenin. In the present case, the neoplasm was positive for S-100, desmin and myogenin, which indicated nerve sheath and rhabdomyoblastic components (2).

Previously published literature has revealed a poor prognosis for MTT. Local recurrence has been observed to be common following tumor excision, while lymphatic invasion and lymph node involvement has not been reported in patients with MTT. Given the rarity of MTT, only case reports and small series of patients have been studied. In 2012, McConnell and Giacomantonio published the largest study to date, including a total of 124 MTT cases from the English and French literature. In the study, the five-year survival rate of MTT was 14%, which was comparable to the previously published five-year survival rate of 11–12%, but significantly worse than the five-year survival rate of other MPNSTs (34–52%). The median survival time of patients with MTT was 13 months, the overall local recurrence rate was 50% and the median time to progression was 6 months. The study suggested that complete surgical resection and adjuvant radiotherapy should be the gold standard treatment for MTT. The study also concluded that conventional chemotherapy does not appear to be of benefit (2).

With regard to location, MTTs occur predominantly in the head, neck and trunk regions. In total, ~20% of MTT cases arise in the head and neck, with 32% in the trunk and 24% in the extremities (15,16). To date, only eight cases located in the mediastinum have been reported, among which, three were in the anterior mediastinum, three in the posterior mediastinum and one in the middle mediastinum (Table I). To the best of our knowledge, this is only the fourth study of an MTT localized in the anterior mediastinum that has been documented in the English literature.

## Figures and Tables

**Table I:** Existing cases reported in the English literature of MTTs located in the mediastinum.

Case/year/ (reference)	Gender/age, years	von Recklinghausen’s disease	Location	Treatment	Recurrence/ residual	Follow-up
1/1984/(4)	F/31	Yes	Anterior mediastinum	Palliative surgery radiotherapy	Yes	Overall survival time, 3 months
2/1984/(5)	M/29	Yes	Posterior mediastinum	No surgery	Yes	Overall survival time, 6 months
3/1985/(3)	F/70	No	Mediastinum	Palliative surgery	Yes	Alive with disease at 53 months
4/1991/(6)	M/39	No	Posterior mediastinum	Palliative surgery chemoradiotherapy	Yes	Overall survival time, 15 months
5/1996/(7)	F/17	Yes	Anterior mediastinum	Palliative surgery radiotherapy	Yes	Overall survival time, 7 months
6/2002/(8)	M/35	Yes	Middle mediastinum	Radical surgery	No	Alive at 18 months
7/2003/(9)	M/22	No	Posterior mediastinum	Radical surgery radiotherapy	No	Alive at 98 months
8/2006/(10)	M/30	No	Anterior mediastinum	Palliative surgery chemoradiotherapy	Yes	Alive with disease at 12 months

MTT, malignant triton tumor.
